# Eplet-Predicted Antigens: An Attempt to Introduce Eplets into Unacceptable Antigen Determination and Calculated Panel-Reactive Antibody Calculation Facilitating Kidney Allocation

**DOI:** 10.3390/diagnostics12122983

**Published:** 2022-11-28

**Authors:** Wenrui Wu, Huanxi Zhang, Jinghong Tan, Qian Fu, Jun Li, Chenglin Wu, Huiting Huang, Bowen Xu, Liuting Ling, Longshan Liu, Xiaojun Su, Changxi Wang

**Affiliations:** 1 Organ Transplant Center, The First Affiliated Hospital, Sun Yat-sen University, Guangzhou 510080, China; 2 Guangdong Provincial Key Laboratory on Organ Donation and Transplant Immunology, Guangzhou 510080, China; 3 Guangdong Provincial International Cooperation Base of Science and Technology (Organ Transplantation), Guangzhou 510080, China

**Keywords:** HLA eplet, unacceptable antigen, calculated panel-reactive antibody, organ allocation, single-antigen bead

## Abstract

(1) Calculated panel-reactive antibody (CPRA) is a measure of sensitization based on unacceptable antigens (UAs). Determination of UAs based on single-antigen bead assays at allele or antigen levels may be inappropriate. We aimed to introduce eplets for better assessment of sensitization; (2) 900 recipients and 1427 donors were enrolled for candidate or donor pools, respectively. Eplets were from the HLA Epitope Registry. UAs were determined by anti-HLA antibodies identified using LIFECODES Single Antigen (LSA) kits. CPRA values were calculated using a simplified method of donor filtering; (3) HLA antigens containing all eplets of an HLA antigen in LSA kits (LSA antigen) were defined as eplet-predicted (EP) antigens, the reactivity of which could be predicted by that LSA antigen. High reactivity concordance was found between LSA and EP antigens. More HLA antigens were covered by EP antigens in the population than LSA antigens. CPRA values at the EP level were higher than at the allele level and lower than at the antigen level. The EP antigens facilitated UA determination for non-LSA antigens and avoided acute rejection; (4) UA determination using EP antigens can lead to more accurate assessment of sensitization, enabling a high probability of compatible organs and a low risk of adverse outcomes.

## 1. Introduction

Kidney transplantation (KT) is an effective renal replacement therapy for patients with end-stage renal disease (ESRD) [1]. Pre-existing donor-specific antibodies (DSA) can lead to hyperacute rejection, antibody-mediated rejection (ABMR) and long-term complications [2]; thus, sensitization is a barrier and candidates with circulating human leucocyte antigen (HLA) antibodies pre-transplantation have decreased access to transplantation [3,4].

Calculated panel-reactive antibody (CPRA) is a measure of sensitization, which is calculated based on unacceptable HLA antigens (UAs) that candidates have been sensitized to and HLA frequencies among the potential donor population. The CPRA value of a candidate represents the percentage of deceased organ donors that express those UAs and will not be offered for that sensitized candidate. The higher the CPRA, the more donor offers would be denied, and additional allocation points would be awarded to highly sensitized candidates to compensate for their longer waiting time [2].

Currently, there is no standard strategy available for the determination of UAs. UAs were traditionally determined at the HLA antigen level based on serum antibody reactivity patterns from HLA panels. With the development of solid-phase bead assays, more and more centers or laboratories used more sensitive assays with single-antigen bead (SAB) kits for HLA antibody detection at the allele level [5]. Determination of UAs at the allele level was shown to facilitate the assessment of DSA and donor selection for sensitized patients [5,6,7,8]. However, although the standard SAB kit was designed to cover the most common HLA alleles [9], with only about 200 beads, it can hardly represent all populations in different regions and cover all HLA antigens present in the population [10]. HLA antigens present in donors but not present in the SAB kits make it difficult to assess the sensitization of candidates directly and accurately [11].

HLA epitopes are a series of polymorphic amino acid (AA) residues of HLA molecules constituting the binding sites of anti-HLA antibodies [12]. Functional residues of epitopes within a 3–3.5 Ångstrom (Å) radius [13,14] that dominate the specificity and strength of binding with anti-HLA antibodies are referred to as eplets [14]. As anti-HLA antibodies are epitope-specific and not allele- or antigen-specific [15], eplets may enable a better understanding of antibody-reactivity patterns of sensitized candidates and more accurate determination of UAs. 

In this study, we introduced a method to determine UAs based on antibody-verified eplets and HLA antibodies detected by SAB kits, aiming at better assessment of sensitization for KT candidates. With UAs determined at different levels, CPRA values were calculated and compared to evaluate their effects on kidney allocation. 

## 2. Materials and Methods

### 2.1. Study Cohort

This single-center retrospective study included patients receiving KT in the First Affiliated Hospital, Sun Yat-sen University, between July 2015 and April 2021. Recipients were enrolled as the candidate pool, and donors were enrolled as the KT donor pool to calculate CPRA values per candidate. Candidates or donors without the second field HLA resolution typing (e.g., A*01:01) were excluded from each pool. A candidate pool with 900 candidates and a donor pool with 1427 donors were included for analysis. 

### 2.2. HLA Typing

All recipients and donors were molecularly typed for HLA-A, -C, -B, -DRB1, and -DQB1 alleles using the Luminex platform and LIFECODES HLA-SSO Typing kit (Immucor Transplant Diagnostics, Stamford, CT, USA) as instructed by the manufacturer. Specific sequences were analyzed using MATCHIT!^TM^ DNA software (version 1.2, Immucor GTI Diagnostics) to determine HLA genotypes.

### 2.3. Anti-HLA Antibodies

Anti-HLA antibodies, including antibodies against HLA-A, -C, -B, -DRB1, and -DQB1 alleles, were detected using the Luminex platform and LIFECODES Single Antigen (LSA) kit (Immucor Transplant Diagnostics, Stamford, CT, USA) as instructed by the manufacturer. Data were analyzed using the LIFECODES MATCHIT!^TM^ ANTIBODY software (version 1.2, Immucor Transplant Diagnostics). A mean fluorescence intensity (MFI) of more than 1000 was considered positive.

### 2.4. Antibody-Verified Eplets

Association between eplets and HLA alleles were obtained from the online HLA Epitope Registry database [http://www.epregistry.com.br/index/databases/database/ (accessed on 16 May 2022)], which documented all theoretically defined HLA eplets based on stereochemical structure, as well as their antibody-verification status [15]. Only antibody-verified eplets were included in our study.

### 2.5. CPRA Calculation and Comparison

UAs were determined based on HLA antibodies of candidates detected by LSA kits at both the allele level (e.g., A*01:01) and antigen level (e.g., A1). HLA antigens of candidates at the allele level were considered acceptable and were not listed as UAs. Unacceptable antigens equivalencies were considered according to the Organ Procurement and Transplantation Network (OPTN) policies document [16]. A simplified method of donor filtering was used for the CPRA calculation at different UA levels [17], where donors were filtered out if the candidate had unacceptable antigens against their HLA antigens. The CRPA value was calculated as the percentage of the number of filtered-out donors over the total number of donors, representing the possibility that a donor was incompatible with the candidate. CPRA values, reclassified categories, and allocation points were compared between different levels using the Student’s *t*-test or Wilcoxon signed-rank test as appropriate.

## 3. Results

### 3.1. LSA Kits Covered Few HLA Antigens in the Donor Population

**Figure 1:**
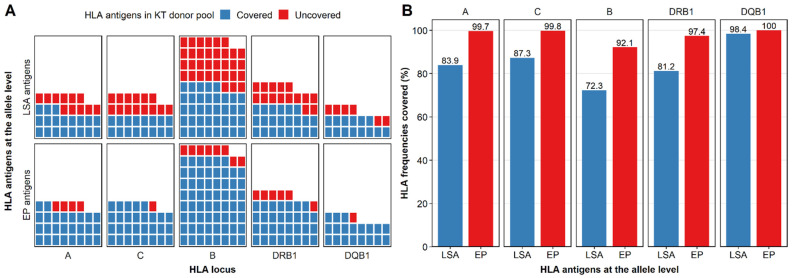
Comparison of donor HLA antigens covered by HLA antigens in the LIFECODES Single Antigen (LSA) kits (LSA antigens) and eplet-predicted antigens (EP antigens). (**A**) Counts of donor HLA antigens covered. Each tile represented an HLA antigen. HLA antigens covered and uncovered by LSA antigens or EP antigens were colored red and blue. (**B**) Frequencies of donor HLA alleles covered by LSA antigens (blue) and EP antigens (red).

The standard LSA kits consisted of 96 beads for class I HLA (30 for the A locus, 48 for the B locus, and 18 for the C locus) and 96 beads for class II HLA (32 for the DRB1 locus with 32 alleles, 31 for the DQA1/DQB1 locus with 14 DQB1 alleles, and 27 for the DPA1/DPB1 locus). There were 187 HLA antigens at the allele level in the KT donor pool, among which antigens in the LSA kits (LSA antigens) covered 57.8% (108/187) of total HLA antigens, including 63.3% (19/30), 53.3% (16/30), 52.9% (37/70), 59.5% (22/37), and 70.0% (14/20) for the HLA-A, -C, -B, -DRB1, and -DQB1 loci, respectively (Figure 1A). These LSA antigens covered 83.9%, 87.3%, 72.3%, 81.2%, and 98.4% of gene frequencies for the HLA-A, -C, -B, -DRB1, and -DQB1 loci, respectively (Figure 1B).

### 3.2. Reactivity of Non-LSA Antigens Could Be Predicted by LSA Antigens Based on Eplets

**Figure 2:**
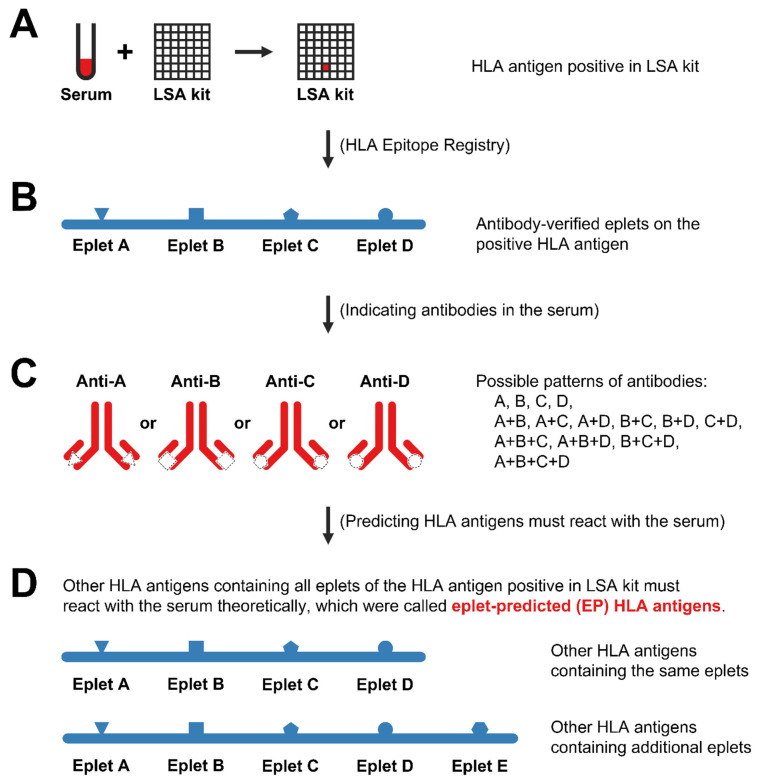
Theory for eplet-predicted (EP) HLA antigens. (**A**) A given serum was added to a LIFECODES Single Antigen (LSA) kit. Antibodies in the serum specifically bind to a bead embedded with single HLA antigens. The reactive HLA antigen was reported as positive. (**B**) Antibody-verified eplets of the positive HLA antigen were documented on the HLA Epitope Registry database. (**C**) As antibodies are eplet-specific, there must be antibodies in the serum that bind to at least one eplet of the positive HLA antigens. However, it is difficult to know the pattern of antibodies and determine which eplets the antibodies specifically react with in a clinical setting. (**D**) If other HLA antigens contain all eplets of the positive LSA antigen (containing either the same eplets or additional eplets), the serum containing antibodies that react with at least one eplet must theoretically react with these HLA antigens. These HLA antigens containing all eplets were called eplet-predicted antigens of the positive HLA antigens. In this way, we conservatively predicted the HLA antigens that must react with a given serum based on the result of LSA kits and eplets.

To predict the antibody reactivity of HLA antigens that were not included in the LSA kits (non-LSA antigens), we used antibody-verified eplets to expand the LSA kits and use LSA antigens to represent the non-LSA antigens. When antibodies in serum react with an LSA antigen, the serum must contain antibodies that react with at least one eplet of this LSA antigen. If an HLA antigen contains all eplets of that positive LSA antigen (containing either the same eplets or additional eplets), the antibodies in serum should theoretically react with this HLA antigen (Figure 2). We then analyzed antibody-verified eplets of all HLA antigens in our KT donor pool or in LSA kits, and predicted which HLA antigens would be reactive if LSA antigens were positive (Table S1). These HLA antigens whose reactivity could be predicted by LSA antigens based on eplets were called eplet-predicted (EP) HLA antigens. For example, A*02:06 and A*02:07 were non-LSA antigens and were found in the KT donor pool. They were EP HLA antigens of A*02:01, A*02:02, and A*02:05 in LSA kits because they had the same antibody-verified eplets (62GE, 62GK, 79GT, 107W, 127K, 144K, 144TKH, 145KHA, 150AAH, 253Q). Likewise, A*24:07 and A*24:10 were EP antigens of A*24:03 because they all had eplets of A*24:03 (62EE, 65GK, 80I, 82LR, 127K, 138MI, 144K, 144KR, 150AAH) and additional eplets (166DG for A*24:07; 163R and 163RW for A*24:10). 

### 3.3. Reactivity Concordance Was Found between LSA Antigens and Their Eplet-Predicted Antigens

**Figure 3:**
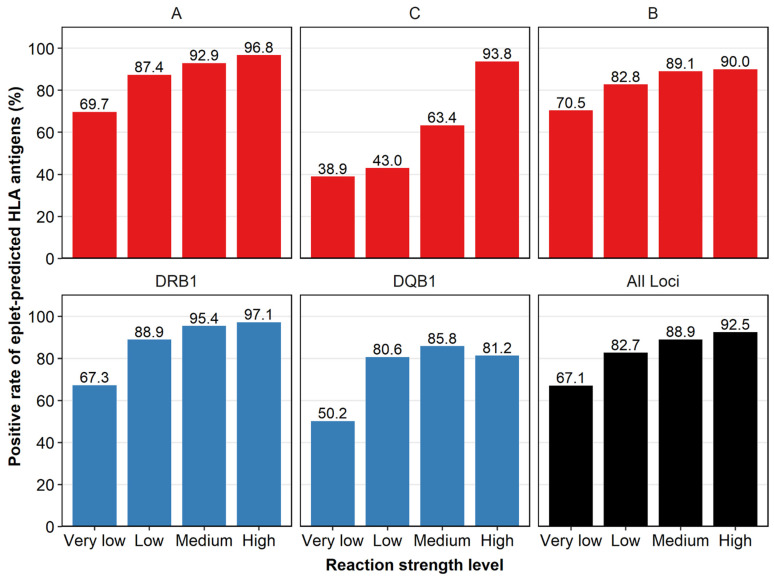
Concordance between LSA antigens and eplet-predicted (EP) HLA antigens at very low (1000–2000), low (2000–4000), medium (4000–10,000), and high (>10,000) reaction strength levels. Positive rates were the percentage of the number of positive over the total number of EP HLA antigens (if they existed in the LSA kits) when the LSA antigens were positive. The higher the reaction strength, the larger the positive rate of EP antigens. The concordance was found at different HLA loci. Class I, class II and all HLA antigens were colored red, blue, and black, respectively.

We compared the reactivity between LSA antigens and their EP antigens present in the LSA kits to see whether they had similar antibody reactivities and were positive at the same time, as we assumed. We divided MFI values into very low (1000–2000), low (2000–4000), medium (4000–10,000), and high (>10,000) reaction strength levels. When an LSA antigen was positive at very low strength, 67.1% (2057/3067) of its EP antigens in the LSA kit (if present) were positive simultaneously. As the reaction strength became higher, the reactivity concordance was higher, with 82.7% (2031/2457), 88.9% (1907/2144), and 92.5% (907/981) of the EP antigens positive at low, medium, and high strength levels, respectively. This reactivity concordance was also found at different HLA loci (Figure 3).

### 3.4. Eplet-Predicted HLA Antigens Covered More HLA Antigens than LSA Kits

Among 187 HLA antigens at the allele level in the KT donor pool, 89.3% (167/187) antigens were covered by EP HLA antigens, higher than the 57.8% (108/187) for LSA antigens. For each HLA locus, EP antigens covered 86.7% (26/30), 96.7% (29/30), 88.6% (62/70), 83.8% (31/37), and 95.0% (19/20) of HLA antigens for HLA-A, -C, -B, -DRB1, and -DQB1 loci, respectively (Figure 1A) and covered 99.7%, 99.8%, 92.1%, 97.4%, and 100.0% of gene frequencies correspondingly (Figure 1B), which were also higher than the LSA kits. 

### 3.5. CPRA (Eplet-Predicted) Were Higher than CPRA (Allele) and Lower than CPRA (Antigen) Generally

Based on the positive HLA antigen in the LSA kits, we determined UAs at the allele level as UAs (allele) and antigen level as UAs (antigen). We also list positive LSA antigens and their EP HLA antigens at the eplet-predicted level as UAs (EP). CPRA values were calculated as CPRA (allele), CPRA (antigen), and CPRA (EP) based on UAs at different levels. We then compared the CRPA values at different levels with 491 LSA reports of candidates that identified UAs for the HLA-A, -C, -B, -DRB1, or -DQB1 loci pre-transplantation. 

**Figure 4:**
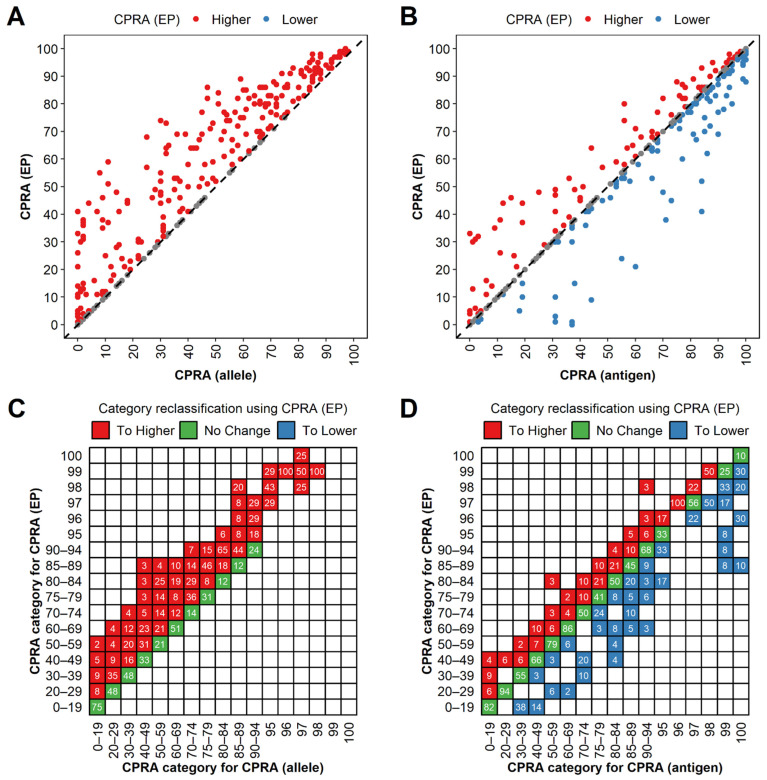
Comparison of calculated panel-reactive antibody (CPRA) values calculated at eplet-predicted level [CPRA (EP)] to those at the HLA allele level [CPRA (allele)] and HLA antigen level [CPRA (antigen)]. (**A**) CPRA (EP) values were higher than CPRA (allele) values for 340 cases (red) and equal for the rest of the cases (grey). (**B**) CPRA (EP) values were higher than CPRA (antigen) values for 121 cases (red) and lower for 170 cases (blue). Reclassification of CPRA categories from CPRA (allele) (**C**) and CPRA (antigen) (**D**) to CPRA (EP). Each case was assigned a primary CPRA category based on the CPRA (allele) or CPRA (antigen) (x-axis) and a secondary CPRA category based on the CPRA (EP) (y-axis). Labels indicate the percentage of cases within the CPRA (allele) or CPRA (antigen) category that moved to different categories using CPRA (EP). Candidates who did not change, moved to higher, or moved to lower categories were colored green, red, or blue, respectively.

The CPRA (EP) was higher than the CPRA (allele) for 340 (340/491, 69.2%) cases and equal for the rest of the cases (Figure 4A). The CPRA (EP) was lower for 170 (170/491, 34.6%) cases and higher than the CPRA (antigen) for 121 (121/491, 24.6%) cases (Figure 4B). Generally, CPRA (EP) values were higher than CPRA (allele) values (*p* < 0.001), and lower than CPRA (antigen) values (*p* = 0.019). 

We then assessed the effects of using the CPRA (EP) as compared to the CPRA (antigen) and CPRA (allele) to assign candidates to CPRA categories in the Kidney Allocation System (KAS). Compared to CPRA (allele), we found that 243 (243/491, 49.5%) cases of CPRA (EP) did not change CPRA categories, whereas 248 (248/491, 50.5%) cases moved to higher categories (Figure 4C). In contrast, compared to CPRA (antigen), 326 (326/491, 66.4%) cases of CPRA (EP) did not change CPRA categories, whereas 92 (92/491, 18.7%) cases moved to lower categories and 73 (73/491, 14.9%) cases moved to higher categories (Figure 4D). The percentage of cases in a category [defined by CPRA (antigen)] that were reclassified to other CPRA categories [defined by CPRA (EP)] varied widely between CPRA categories, from a low of 6% for the 20–29% category to 100% for the 96% category (Figure 4D). When calculating the CPRA at the EP level, among 17 categories, there were fewer categories (5/17, 29.4%; 0–19%, 20–29%, 50–59%, 75–79%, and 96% categories) which had more cases reclassified to higher categories. The magnitude of category reclassification was smaller when moving to higher categories than to lower categories using the CPRA (EP). Among 73 cases that moved to higher categories, 39 (53.4%), 27 (40.0%), 5 (6.8%), and 2 (2.7%) cases moved to one, two, three, and four higher categories, respectively. By comparison, among 92 cases that moved to lower categories, 33 (35.9%), 31 (33.7%), 15 (16.3%), 8 (8.7%), 5 (5.4%) cases moved to one, two, three, four, and more than four lower categories, respectively. Sensitized candidates could gain more access to kidney transplantation when UAs were determined at the EP level, with generally lower CRPA reclassified categories (*p* = 0.003) and fewer allocation points (*p* < 0.001), compared to determining UAs at the antigen level.

### 3.6. Eplet-Predicted Antigens Facilitated UA Determination for Non-LSA Antigens and Avoided Acute Rejection

To visually illustrate the benefits of EP antigens intuitively, we presented a real-world clinical case as an example. A 42-year-old male kidney transplant candidate had anti-HLA antibodies against A*02:05 (MFI = 5545), A*02:02 (MFI = 2890), A*02:01 (MFI = 1870), and other HLA antigens at other loci. A kidney typed as A*02:06 and A*03:01 was transplanted to this candidate with consideration that the LSA kit did not report a positive result for A*02:06. Soon after he received the KT operation, a significant increase in the MFI of A*02:05 (15810), A*02:02 (14117) and A*02:01 (8994), and delayed graft function (DGF) were observed. Increased resistivity index (about 1.0) and ischemia foci were identified by Doppler ultrasound. This patient was diagnosed with acute rejection and treated with methylprednisolone pulse therapy for three days. Retrospectively, A*02:06 was not included in LSA kits and would never be reported as positive, which made it difficult to decide whether to list A*02:06 as a UA at the allele level. However, A*02:06 was an EP antigen of A*02:01, A*02:02, and A*02:05, with the same antibody-verified eplets (62GE, 62GK, 79GT, 107W, 127K, 144K, 144TKH, 145KHA, 150AAH, and 253Q), indicating that these HLA antigens had similar antibody reactivities and should be listed as UAs. Donor kidneys typed as A*02:06 would be denied, and acute rejection could be avoided.

## 4. Discussion

Candidates of solid organ transplantation on the waiting list are at risk of adverse outcomes due to high sensitization. The higher the CPRA, the lower the percentage of candidates who receive a transplant and the higher the percentage of candidates who die [18,19,20,21]. Clinicians should be highly cautious when selecting UAs to reduce the CPRA as much as possible. However, over-lenient criteria for UA determination to reduce CPRA values is not appropriate. Without assigning sensitized antigens as UAs, the probability of a positive crossmatch (XM) may increase, which can lead to decreased efficiency of organ allocation and increased risk of graft failure because of additional cold ischemia time [22,23,24]. Furthermore, receiving a graft that the candidate is sensitized to is associated with hyperacute rejection, DGF, and long-term complications. These circumstances would lead to a poor prognosis post-transplantation and even more sensitized candidates in the future, which is a waste of scarce donor resources [25,26,27,28]. Therefore, an appropriate strategy for the determination of UAs is of great importance. Sensitization was not associated with adverse outcomes when UAs were assigned accordingly, with potentially reactive HLA antibodies avoided [29].

UAs were generally determined at the HLA antigen level and allele level. Determining UAs at the antigen level usually overestimates CPRA values because antibodies against HLA antigens are sometimes against specific HLA alleles within this antigen group [30]. When we list UAs at the antigen level and exclude all alleles within these antigen groups, the probability of finding compatible donors will decrease. Assigning UAs at the allele level was shown to improve the accuracy of virtual crossmatching, allow correct assessment of HLA sensitization, and increase the probability of finding immunologically compatible donors [6,7,31,32]. However, with limited beads in LSA kits, only a small number of HLA antigens (57.8% in our study) was covered in the reference population [10], making it difficult to determine UAs at the allele level directly based on the result of LSA assays. Potential DSA could be omitted, and CPRA values would then be underestimated when determining UAs at the allele level, which may lead to a higher risk of XM and hyperacute rejection. 

In our study, we developed a new method for better determination of UAs. Based on the reactive HLA antigens revealed by LSA kits and antibody-verified eplets of them, we predicted the HLA antigens that would theoretically react with antibodies in the serum of candidates but which were not indicated by LSA kits directly (non-LSA antigens). Anti-HLA antibodies are eplet-specific rather than antigen- or allele-specific [15,33], but it was challenging to figure out exactly which eplet reacted with the antibodies in a clinical setting. Therefore, we applied a conservative strategy to predict which HLA antigens would react with the serum. When antibodies in serum react with beads embedded with single HLA antigens in the LSA kits, the serum must contain antibodies that react with at least one eplet of this HLA antigen. Because its EP antigens have all eplets of this antigen, including the one that reacts with antibodies, the reactivity of HLA antigens can theoretically represent the reactivity of their EP antigens. Given that most eplets in the HLA Epitope Registry database remain theoretical and are not intended for clinical decision-making [9], only eplets verified by antibodies were included in our study [34,35]. Our method was conservative to avoid an overly high CPRA with a limited probability of compatible organs, but which would be more accurate to reduce the risk of XM and other adverse outcomes. 

As it was difficult to compare reactivity between LSA antigens and their EP antigens which are non-LSA antigens, we compared antibody reactivity between LSA antigens and their EP antigens if they were all in the LSA kits. High reactivity concordance was observed in our study; LSA antigens and their EP antigens seemed to share the same antibody reactivities and be positive simultaneously, as we expected. At a low reaction strength level (MFI < 2000), the concordance was relatively low, which may be explained by the shared epitope phenomenon (dilution effect) [36,37]. An antibody reacting with one of eplets will bind to all the antigens that express it in LSA kits, diluting the antibodies that bind to each bead. Because the MFI is measured per bead in LSA kits, the reaction strength of the antibodies may be underestimated, leading to false negative results and, therefore, unexpected positive physical XMs [11]. The stronger the reaction strength, the weaker the effect of antibody dilution for each bead [38]. This was consistent with our study showing that with increasing MFI values, LSA antigens and their EP antigens tended to be positive simultaneously in LSA kits, which could be masked by the dilution effect at low reaction strength level. At a relatively low MFI value (>2000), reactivity concordance was acceptable (about 85–90%) for the HLA-A, -B, and -DRB1 loci. This result indicated a similar antibody reactivity between HLA antigens and their EP antigens which validated our method for expanding UAs by adding EP antigens of the positive LSA antigens as UAs. However, a relatively low concordance was observed for the HLA-C and -DQB1 loci, which may be due to the small number of antibody-verified eplets for the HLA-C and -DQB1 loci on the HLA Epitope Registry database (Table S2). With more and more eplets being antibody-verified experimentally, our method for UA determination can be more accurate, and higher concordance of antibody reactivities between LSA antigens and their EP antigens is foreseeable in the future [34,35]. 

One of the benefits of EP antigens was the assessment of sensitization for non-LSA antigens. Based on eplets, antibody reactivities of the non-LSA antigens can be represented by LSA antigens. Similar to other studies [10], only a small proportion of HLA antigens (57.8% in our study) and donor population (80–85% of gene frequencies in our study) were directly covered by LSA kits because of the diversity of HLA allelic distribution between different areas and ethnic groups [39,40,41]. With the reactivity of EP antigens represented by LSA antigens, about 90% of HLA antigens and over 95% of the donor population were covered, meaning that we can determine UAs more accurately for most cases with this method. In our case, when we determined UAs at the allele level, it was difficult to list A*02:06 and A*02:07 as UAs because these alleles were not included in the LSA kits. These non-SAB alleles, A*02:06 and A*02:07, covered a large proportion of the KT donor population (27.6%, 396/1437) in our center. By assigning these non-LSA antigens as UAs, we could effectively avoid positive XMs and adverse outcomes such as hyperacute or acute rejection caused by pre-existing DSA. In addition to a more accurate assessment of sensitization, determining UAs based on EP antigens enabled generally low CPRA values in kidney allocation. CPRA (EP) values were lower than those of CPRA (antigen) (*p* = 0.019), especially for highly sensitized candidates; these candidates would be reclassified into lower categories (*p* = 0.002) and receive less allocation (*p* < 0.001), indicating a relatively higher probability of receiving compatible kidneys. Overall, our method for CPRA calculation by expanding UAs with antibody-verified eplets facilitated kidney allocation and enabled a balance between a relatively high probability of receiving allograft (compared to CPRA at the antigen level) and a relatively low risk of adverse outcomes post-transplantation (compared to CPRA at the allele level). 

Despite these advantages, several limitations need consideration. Firstly, our study was a single-center retrospective study, and CRPA values were calculated based on the KT donor pool of our center, which may be different from those calculated based on other populations. Secondly, more evidence is needed to prove that the reactivity of EP antigens can be represented by LSA antigens as we predicted. Concordance between LSA antigens and non-LSA antigens may be verified by further studies based on results of crossmatching. Thirdly, only a small amount of eplets defined theoretically have been validated by antibody verification. Although antibody-verified eplets mismatch has been shown to be associated with DSA formation and graft survival, eplets that have not been antibody-verified were also indicated to be clinically relevant [42,43,44], which may lead to inaccurate expansion of LSA antigens based on antibody-verified eplets. But with more and more eplets verified by further experiments, our method would be more practical and reliable. Lastly, MFI thresholds used to determine positive results are different between different centers, with cutoff values ranging between 500 and 3000 in other studies [45,46,47,48]. In our study, we used an arbitrary MFI threshold value of 1000. We also performed the same analysis using higher MFI threshold values (2000 and 3000), which did not change the overall tendency and concept of our study. 

## 5. Conclusions

In conclusion, we propose a concept of eplet-predicted antigens based on antibody-verified eplets and HLA antibodies detected by LSA kits. Concordance of antibody reactivity was shown between LSA antigens and their EP antigens in LSA kits. Based on this method, we extended the universe of UAs beyond those indicated by LSA kits and allowed more accurate determination of UAs for CPRA calculation. Our method of expanding UAs with EP antigens based on eplets is a new attempt to introduce eplets into organ allocation, aiming to keep a balance between a relatively high probability of compatible organs and a relatively low risk of adverse outcomes caused by pre-existing DSA. 

The introduction of eplets analysis into allocation consideration and an allocation system based on eplets matching, while appealing, is premature [49]. However, our method is a supplement to the current allocation system, which does not change the equity and accountability of the current system and is ready for immediate use by adding EP antigens of previously determined UAs as UAs. It should be noted that EP antigens of LSA antigens are not unchangeable but change with updates of the eplets database. It is foreseeable that with more eplets verified by antibodies experimentally and a better understanding of eplets, a better reactivity concordance could be achieved, and our method would be more reliable and accurate for sensitization assessment in the future.

## Data Availability

Not applicable.

## Supplementary Materials

The following supporting information can be downloaded at: https://www.mdpi.com/article/10.3390/diagnostics12122983/s1, Table S1: LSA antigens and their eplet-predicted antigens; Table S2: Numbers of antibody-verified eplets shared by LSA antigens and their eplet-predicted antigens.
