# Supplementary material for: Eplet-Predicted Antigens: An Attempt to Introduce Eplets into Unacceptable Antigen Determination and Calculated Panel-Reactive Antibody Calculation Facilitating Kidney Allocation
Source: Diagnostics (Basel). 2022 Nov 28;12(12):2983. doi: 10.3390/diagnostics12122983 (PMC9776513; doi:10.3390/diagnostics12122983)
Supplement: Supplementary file 1 [file diagnostics-12-02983-s001.zip › diagnostics-1962650-supplementary.pdf]

Table S1. LSA antigens and their eplet-predicted antigens.

LSA antigens	Eplet-predicted antigens	Eplet abbreviate of eplet-predicted antigens	Eplets of LSA antigens	Eplets of eplet-predicted antigens
A*01:01	A*01:01	A*01:01	44KM, 62QE, 65RNA, 76ANT, 79GT, 90D, 138MI, 144K, 144KR, 163R, 163RG, 166DG	44KM, 62QE, 65RNA, 76ANT, 79GT, 90D, 138MI, 144K, 144KR, 163R, 163RG, 166DG
A*02:01	A*02:01, A*02:02, A*02:05, A*02:06, A*02:07	A*02:01	62GE, 62GK, 79GT, 107W, 127K, 144K, 144TKH, 145KHA, 150AAH, 253Q	62GE, 62GK, 79GT, 107W, 127K, 144K, 144TKH, 145KHA, 150AAH, 253Q
A*02:02	A*02:01, A*02:02, A*02:05, A*02:06, A*02:07	A*02:02	62GE, 62GK, 79GT, 107W, 127K, 144K, 144TKH, 145KHA, 150AAH, 253Q	62GE, 62GK, 79GT, 107W, 127K, 144K, 144TKH, 145KHA, 150AAH, 253Q
A*02:03	A*02:03	A*02:03	62GE, 62GK, 79GT, 107W, 127K, 144K, 144TKH, 149TAH, 253Q	62GE, 62GK, 79GT, 107W, 127K, 144K, 144TKH, 149TAH, 253Q
A*02:05	A*02:01, A*02:02, A*02:05, A*02:06, A*02:07	A*02:05	62GE, 62GK, 79GT, 107W, 127K, 144K, 144TKH, 145KHA, 150AAH, 253Q	62GE, 62GK, 79GT, 107W, 127K, 144K, 144TKH, 145KHA, 150AAH, 253Q
A*03:01	A*03:01, A*03:02	A*03:01	62QE, 65RNA, 79GT, 138MI, 144K, 144KR, 150AAH, 161D	62QE, 65RNA, 79GT, 138MI, 144K, 144KR, 150AAH, 161D
A*11:01	A*11:01, A*11:02	A*11:01	62QE, 65RNA, 79GT, 90D, 138MI, 144K, 144KR, 150AAH, 151AHA, 163R, 163RW	62QE, 65RNA, 79GT, 90D, 138MI, 144K, 144KR, 150AAH, 151AHA, 163R, 163RW
A*11:02	A*11:01, A*11:02	A*11:02	62QE, 65RNA, 79GT, 90D, 138MI, 144K, 144KR, 150AAH, 151AHA, 163R, 163RW	62QE, 65RNA, 79GT, 90D, 138MI, 144K, 144KR, 150AAH, 151AHA, 163R, 163RW
A*23:01	A*23:01	A*23:01	62EE, 65GK, 80I, 82LR, 127K, 138MI, 166DG	62EE, 65GK, 80I, 82LR, 127K, 138MI, 166DG
A*23:01	A*24:02, A*24:07	A*23:01 + 144K + 144KR + 150AAH	62EE, 65GK, 80I, 82LR, 127K, 138MI, 166DG	62EE, 65GK, 80I, 82LR, 127K, 138MI, 144K, 144KR, 150AAH, 166DG
A*24:02	A*24:02, A*24:07	A*24:02	62EE, 65GK, 80I, 82LR, 127K, 138MI, 144K, 144KR, 150AAH, 166DG	62EE, 65GK, 80I, 82LR, 127K, 138MI, 144K, 144KR, 150AAH, 166DG

A*24:03	A*24:02, A*24:07	A*24:03 + 166DG	62EE, 65GK, 80I, 82LR, 127K, 138MI, 144K, 144KR, 150AAH	62EE, 65GK, 80I, 82LR, 127K, 138MI, 144K, 144KR, 150AAH, 166DG
A*24:03	A*24:03	A*24:03	62EE, 65GK, 80I, 82LR, 127K, 138MI, 144K, 144KR, 150AAH	62EE, 65GK, 80I, 82LR, 127K, 138MI, 144K, 144KR, 150AAH
A*24:03	A*24:10	A*24:03 + 163R + 163RW	62EE, 65GK, 80I, 82LR, 127K, 138MI, 144K, 144KR, 150AAH	62EE, 65GK, 80I, 82LR, 127K, 138MI, 144K, 144KR, 150AAH, 163R, 163RW
A*25:01	A*25:01	A*25:01	62RR, 65RNA, 76ESI, 80I, 82LR, 90D, 138MI, 145RT, 149TAH, 163R, 163RW, 253Q	62RR, 65RNA, 76ESI, 80I, 82LR, 90D, 138MI, 145RT, 149TAH, 163R, 163RW, 253Q
A*26:01	A*26:01	A*26:01	62RR, 65RNA, 76ANT, 79GT, 90D, 138MI, 145RT, 149TAH, 163R, 163RW, 253Q	62RR, 65RNA, 76ANT, 79GT, 90D, 138MI, 145RT, 149TAH, 163R, 163RW, 253Q
A*29:01	A*29:01, A*29:02	A*29:01	62LQ, 65RNA, 76ANT, 79GT, 138MI, 253Q	62LQ, 65RNA, 76ANT, 79GT, 138MI, 253Q
A*29:01	A*43:01	A*29:01 + 90D + 145RT + 149TAH + 163R + 163RW	62LQ, 65RNA, 76ANT, 79GT, 138MI, 253Q	62LQ, 65RNA, 76ANT, 79GT, 90D, 138MI, 145RT, 149TAH, 163R, 163RW, 253Q
A*29:02	A*29:01, A*29:02	A*29:02	62LQ, 65RNA, 76ANT, 79GT, 138MI, 253Q	62LQ, 65RNA, 76ANT, 79GT, 138MI, 253Q
A*29:02	A*43:01	A*29:02 + 90D + 145RT + 149TAH + 163R + 163RW	62LQ, 65RNA, 76ANT, 79GT, 138MI, 253Q	62LQ, 65RNA, 76ANT, 79GT, 90D, 138MI, 145RT, 149TAH, 163R, 163RW, 253Q
A*30:01	A*30:01	A*30:01	56R, 62QE, 65RNA, 79GT, 138MI	56R, 62QE, 65RNA, 79GT, 138MI
A*30:01	A*30:04	A*30:01 + 76EG + 150AAH	56R, 62QE, 65RNA, 79GT, 138MI	56R, 62QE, 65RNA, 76EG, 79GT, 138MI, 150AAH
A*30:01	A*31:01	A*30:01 + 253Q	56R, 62QE, 65RNA, 79GT, 138MI	56R, 62QE, 65RNA, 79GT, 138MI, 253Q
A*31:01	A*31:01	A*31:01	56R, 62QE, 65RNA, 79GT, 138MI, 253Q	56R, 62QE, 65RNA, 79GT, 138MI, 253Q
A*32:01	A*32:01	A*32:01	62QE, 65RNA, 76ESI, 80I, 82LR, 138MI, 253Q	62QE, 65RNA, 76ESI, 80I, 82LR, 138MI, 253Q

A*33:01	A*26:01	A*33:01 + 76ANT + 90D + 145RT + 149TAH + 163R + 163RW	62RR, 65RNA, 79GT, 138MI, 253Q	62RR, 65RNA, 76ANT, 79GT, 90D, 138MI, 145RT, 149TAH, 163R, 163RW, 253Q
A*33:01	A*33:01, A*33:03	A*33:01	62RR, 65RNA, 79GT, 138MI, 253Q	62RR, 65RNA, 79GT, 138MI, 253Q
A*33:01	A*34:02	A*33:01 + 90D + 145RT + 149TAH	62RR, 65RNA, 79GT, 138MI, 253Q	62RR, 65RNA, 79GT, 90D, 138MI, 145RT, 149TAH, 253Q
A*33:01	A*66:01	A*33:01 + 90D + 145RT + 149TAH + 163R + 163RW	62RR, 65RNA, 79GT, 138MI, 253Q	62RR, 65RNA, 79GT, 90D, 138MI, 145RT, 149TAH, 163R, 163RW, 253Q
A*33:01	A*66:02	A*33:01 + 145RT + 149TAH + 163EW	62RR, 65RNA, 79GT, 138MI, 253Q	62RR, 65RNA, 79GT, 138MI, 145RT, 149TAH, 163EW, 253Q
A*33:03	A*26:01	A*33:03 + 76ANT + 90D + 145RT + 149TAH + 163R + 163RW	62RR, 65RNA, 79GT, 138MI, 253Q	62RR, 65RNA, 76ANT, 79GT, 90D, 138MI, 145RT, 149TAH, 163R, 163RW, 253Q
A*33:03	A*33:01, A*33:03	A*33:03	62RR, 65RNA, 79GT, 138MI, 253Q	62RR, 65RNA, 79GT, 138MI, 253Q
A*33:03	A*34:02	A*33:03 + 90D + 145RT + 149TAH	62RR, 65RNA, 79GT, 138MI, 253Q	62RR, 65RNA, 79GT, 90D, 138MI, 145RT, 149TAH, 253Q
A*33:03	A*66:01	A*33:03 + 90D + 145RT + 149TAH + 163R + 163RW	62RR, 65RNA, 79GT, 138MI, 253Q	62RR, 65RNA, 79GT, 90D, 138MI, 145RT, 149TAH, 163R, 163RW, 253Q
A*33:03	A*66:02	A*33:03 + 145RT + 149TAH + 163EW	62RR, 65RNA, 79GT, 138MI, 253Q	62RR, 65RNA, 79GT, 138MI, 145RT, 149TAH, 163EW, 253Q
A*34:02	A*26:01	A*34:02 + 76ANT + 163R + 163RW	62RR, 65RNA, 79GT, 90D, 138MI, 145RT, 149TAH, 253Q	62RR, 65RNA, 76ANT, 79GT, 90D, 138MI, 145RT, 149TAH, 163R, 163RW, 253Q
A*34:02	A*34:02	A*34:02	62RR, 65RNA, 79GT, 90D, 138MI, 145RT, 149TAH, 253Q	62RR, 65RNA, 79GT, 90D, 138MI, 145RT, 149TAH, 253Q
A*34:02	A*66:01	A*34:02 + 163R + 163RW	62RR, 65RNA, 79GT, 90D, 138MI, 145RT, 149TAH, 253Q	62RR, 65RNA, 79GT, 90D, 138MI, 145RT, 149TAH, 163R, 163RW, 253Q

A*36:01	A*01:01	A*36:01 + 163R + 163RG + 166DG	44KM, 62QE, 65RNA, 76ANT, 79GT, 90D, 138MI, 144K, 144KR	44KM, 62QE, 65RNA, 76ANT, 79GT, 90D, 138MI, 144K, 144KR, 163R, 163RG, 166DG
A*36:01	A*36:01	A*36:01	44KM, 62QE, 65RNA, 76ANT, 79GT, 90D, 138MI, 144K, 144KR	44KM, 62QE, 65RNA, 76ANT, 79GT, 90D, 138MI, 144K, 144KR
A*43:01	A*43:01	A*43:01	62LQ, 65RNA, 76ANT, 79GT, 90D, 138MI, 145RT, 149TAH, 163R, 163RW, 253Q	62LQ, 65RNA, 76ANT, 79GT, 90D, 138MI, 145RT, 149TAH, 163R, 163RW, 253Q
A*66:01	A*26:01	A*66:01 + 76ANT	62RR, 65RNA, 79GT, 90D, 138MI, 145RT, 149TAH, 163R, 163RW, 253Q	62RR, 65RNA, 76ANT, 79GT, 90D, 138MI, 145RT, 149TAH, 163R, 163RW, 253Q
A*66:01	A*66:01	A*66:01	62RR, 65RNA, 79GT, 90D, 138MI, 145RT, 149TAH, 163R, 163RW, 253Q	62RR, 65RNA, 79GT, 90D, 138MI, 145RT, 149TAH, 163R, 163RW, 253Q
A*66:02	A*66:02	A*66:02	62RR, 65RNA, 79GT, 138MI, 145RT, 149TAH, 163EW, 253Q	62RR, 65RNA, 79GT, 138MI, 145RT, 149TAH, 163EW, 253Q
A*68:01	A*68:01, A*68:02	A*68:01	62RR, 65RNA, 79GT, 127K, 144K, 144TKH, 145KHA, 150AAH, 253Q	62RR, 65RNA, 79GT, 127K, 144K, 144TKH, 145KHA, 150AAH, 253Q
A*68:01	A*69:01	A*68:01 + 107W	62RR, 65RNA, 79GT, 127K, 144K, 144TKH, 145KHA, 150AAH, 253Q	62RR, 65RNA, 79GT, 107W, 127K, 144K, 144TKH, 145KHA, 150AAH, 253Q
A*68:02	A*68:01, A*68:02	A*68:02	62RR, 65RNA, 79GT, 127K, 144K, 144TKH, 145KHA, 150AAH, 253Q	62RR, 65RNA, 79GT, 127K, 144K, 144TKH, 145KHA, 150AAH, 253Q
A*68:02	A*69:01	A*68:02 + 107W	62RR, 65RNA, 79GT, 127K, 144K, 144TKH, 145KHA, 150AAH, 253Q	62RR, 65RNA, 79GT, 107W, 127K, 144K, 144TKH, 145KHA, 150AAH, 253Q
A*69:01	A*69:01	A*69:01	62RR, 65RNA, 79GT, 107W, 127K, 144K, 144TKH, 145KHA, 150AAH, 253Q	62RR, 65RNA, 79GT, 107W, 127K, 144K, 144TKH, 145KHA, 150AAH, 253Q
A*74:01	A*31:01	A*74:01 + 56R	62QE, 65RNA, 79GT, 138MI, 253Q	56R, 62QE, 65RNA, 79GT, 138MI, 253Q
A*74:01	A*74:01, A*74:02	A*74:01	62QE, 65RNA, 79GT, 138MI, 253Q	62QE, 65RNA, 79GT, 138MI, 253Q

A*80:01	A*80:01	A*80:01	62EE, 65RNA, 76ANT, 79GT, 90D, 138MI, 144K, 144KR, 166DG	62EE, 65RNA, 76ANT, 79GT, 90D, 138MI, 144K, 144KR, 166DG
B*07:02	B*07:02, B*07:05	B*07:02	65QIA, 69AA, 70IAQ, 76ESN, 80N, 163EW, 180E	65QIA, 69AA, 70IAQ, 76ESN, 80N, 163EW, 180E
B*07:02	B*81:01	B*07:02 + 143S	65QIA, 69AA, 70IAQ, 76ESN, 80N, 163EW, 180E	65QIA, 69AA, 70IAQ, 76ESN, 80N, 143S, 163EW, 180E
B*07:03	B*07:03	B*07:03	69TNT, 71TTS, 76ESN, 80N, 163EW, 180E	69TNT, 71TTS, 76ESN, 80N, 163EW, 180E
B*07:03	B*40:01	B*07:03 + 41T + 45KE + 143S	69TNT, 71TTS, 76ESN, 80N, 163EW, 180E	41T, 45KE, 69TNT, 71TTS, 76ESN, 80N, 143S, 163EW, 180E
B*07:03	B*48:01, B*48:03	B*07:03 + 143S	69TNT, 71TTS, 76ESN, 80N, 163EW, 180E	69TNT, 71TTS, 76ESN, 80N, 143S, 163EW, 180E
B*08:01	B*08:01	B*08:01	69TNT, 71TTS, 76ESN, 80N, 156DA, 180E	69TNT, 71TTS, 76ESN, 80N, 156DA, 180E
B*08:01	B*41:01	B*08:01 + 41T + 45KE	69TNT, 71TTS, 76ESN, 80N, 156DA, 180E	41T, 45KE, 69TNT, 71TTS, 76ESN, 80N, 156DA, 180E
B*13:02	B*13:01, B*13:02	B*13:02	41T, 44RMA, 69TNT, 80TLR, 82LR, 131S, 144QL, 163EW	41T, 44RMA, 69TNT, 80TLR, 82LR, 131S, 144QL, 163EW
B*14:01	B*14:01, B*14:02	B*14:01	69TNT, 71TTS, 76ESN, 80N, 131S	69TNT, 71TTS, 76ESN, 80N, 131S
B*14:01	B*15:01, B*15:02, B*15:05, B*15:07, B*15:11, B*15:21, B*15:25, B*15:27, B*15:32	B*14:01 + 44RMA + 163LW	69TNT, 71TTS, 76ESN, 80N, 131S	44RMA, 69TNT, 71TTS, 76ESN, 80N, 131S, 163LW
B*14:01	B*15:03, B*15:18	B*14:01 + 163LW	69TNT, 71TTS, 76ESN, 80N, 131S	69TNT, 71TTS, 76ESN, 80N, 131S, 163LW
B*14:01	B*15:12	B*14:01 + 44RMA + 163LS/G + 166DG	69TNT, 71TTS, 76ESN, 80N, 131S	44RMA, 69TNT, 71TTS, 76ESN, 80N, 131S, 163LS/G, 166DG
B*14:01	B*18:01, B*18:02	B*14:01 + 44RT	69TNT, 71TTS, 76ESN, 80N, 131S	44RT, 69TNT, 71TTS, 76ESN, 80N, 131S
B*14:01	B*35:01, B*35:02, B*35:03, B*35:05, B*35:08, B*78:01	B*14:01 + 44RT + 163LW + 193PV	69TNT, 71TTS, 76ESN, 80N, 131S	44RT, 69TNT, 71TTS, 76ESN, 80N, 131S, 163LW, 193PV

B*14:01	B*39:01, B*39:05, B*39:09, B*39:15	B*14:01 + 158T	69TNT, 71TTS, 76ESN, 80N, 131S	69TNT, 71TTS, 76ESN, 80N, 131S, 158T
B*14:01	B*45:01	B*14:01 + 41T + 45KE + 156DA + 163LS/G	69TNT, 71TTS, 76ESN, 80N, 131S	41T, 45KE, 69TNT, 71TTS, 76ESN, 80N, 131S, 156DA, 163LS/G
B*14:01	B*50:01	B*14:01 + 41T + 45KE + 163LW	69TNT, 71TTS, 76ESN, 80N, 131S	41T, 45KE, 69TNT, 71TTS, 76ESN, 80N, 131S, 163LW
B*14:02	B*14:01, B*14:02	B*14:02	69TNT, 71TTS, 76ESN, 80N, 131S	69TNT, 71TTS, 76ESN, 80N, 131S
B*14:02	B*15:01, B*15:02, B*15:05, B*15:07, B*15:11, B*15:21, B*15:25, B*15:27, B*15:32	B*14:02 + 44RMA + 163LW	69TNT, 71TTS, 76ESN, 80N, 131S	44RMA, 69TNT, 71TTS, 76ESN, 80N, 131S, 163LW
B*14:02	B*15:03, B*15:18	B*14:02 + 163LW	69TNT, 71TTS, 76ESN, 80N, 131S	69TNT, 71TTS, 76ESN, 80N, 131S, 163LW
B*14:02	B*15:12	B*14:02 + 44RMA + 163LS/G + 166DG	69TNT, 71TTS, 76ESN, 80N, 131S	44RMA, 69TNT, 71TTS, 76ESN, 80N, 131S, 163LS/G, 166DG
B*14:02	B*18:01, B*18:02	B*14:02 + 44RT	69TNT, 71TTS, 76ESN, 80N, 131S	44RT, 69TNT, 71TTS, 76ESN, 80N, 131S
B*14:02	B*35:01, B*35:02, B*35:03, B*35:05, B*35:08, B*78:01	B*14:02 + 44RT + 163LW + 193PV	69TNT, 71TTS, 76ESN, 80N, 131S	44RT, 69TNT, 71TTS, 76ESN, 80N, 131S, 163LW, 193PV
B*14:02	B*39:01, B*39:05, B*39:09, B*39:15	B*14:02 + 158T	69TNT, 71TTS, 76ESN, 80N, 131S	69TNT, 71TTS, 76ESN, 80N, 131S, 158T
B*14:02	B*45:01	B*14:02 + 41T + 45KE + 156DA + 163LS/G	69TNT, 71TTS, 76ESN, 80N, 131S	41T, 45KE, 69TNT, 71TTS, 76ESN, 80N, 131S, 156DA, 163LS/G
B*14:02	B*50:01	B*14:02 + 41T + 45KE + 163LW	69TNT, 71TTS, 76ESN, 80N, 131S	41T, 45KE, 69TNT, 71TTS, 76ESN, 80N, 131S, 163LW
B*15:01	B*15:01, B*15:02, B*15:05, B*15:07, B*15:11, B*15:21, B*15:25, B*15:27, B*15:32	B*15:01	44RMA, 69TNT, 71TTS, 76ESN, 80N, 131S, 163LW	44RMA, 69TNT, 71TTS, 76ESN, 80N, 131S, 163LW
B*15:02	B*15:01, B*15:02, B*15:05, B*15:07, B*15:11, B*15:21, B*15:25, B*15:27, B*15:32	B*15:02	44RMA, 69TNT, 71TTS, 76ESN, 80N, 131S, 163LW	44RMA, 69TNT, 71TTS, 76ESN, 80N, 131S, 163LW

B*15:03	B*15:01, B*15:02, B*15:05, B*15:07, B*15:11, B*15:21, B*15:25, B*15:27, B*15:32	B*15:03 + 44RMA	69TNT, 71TTS, 76ESN, 80N, 131S, 163LW	44RMA, 69TNT, 71TTS, 76ESN, 80N, 131S, 163LW
B*15:03	B*15:03, B*15:18	B*15:03	69TNT, 71TTS, 76ESN, 80N, 131S, 163LW	69TNT, 71TTS, 76ESN, 80N, 131S, 163LW
B*15:03	B*35:01, B*35:02, B*35:03, B*35:05, B*35:08, B*78:01	B*15:03 + 44RT + 193PV	69TNT, 71TTS, 76ESN, 80N, 131S, 163LW	44RT, 69TNT, 71TTS, 76ESN, 80N, 131S, 163LW, 193PV
B*15:03	B*50:01	B*15:03 + 41T + 45KE	69TNT, 71TTS, 76ESN, 80N, 131S, 163LW	41T, 45KE, 69TNT, 71TTS, 76ESN, 80N, 131S, 163LW
B*15:12	B*15:12	B*15:12	44RMA, 69TNT, 71TTS, 76ESN, 80N, 131S, 163LS/G, 166DG	44RMA, 69TNT, 71TTS, 76ESN, 80N, 131S, 163LS/G, 166DG
B*15:13	B*15:13	B*15:13	44RMA, 69TNT, 80I, 82LR, 131S, 163LW	44RMA, 69TNT, 80I, 82LR, 131S, 163LW
B*15:16	B*15:16, B*15:17	B*15:16	44RMA, 62RR, 65RNA, 69AA, 71SA, 80I, 82LR, 131S, 163LW	44RMA, 62RR, 65RNA, 69AA, 71SA, 80I, 82LR, 131S, 163LW
B*15:18	B*15:01, B*15:02, B*15:05, B*15:07, B*15:11, B*15:21, B*15:25, B*15:27, B*15:32	B*15:18 + 44RMA	69TNT, 71TTS, 76ESN, 80N, 131S, 163LW	44RMA, 69TNT, 71TTS, 76ESN, 80N, 131S, 163LW
B*15:18	B*15:03, B*15:18	B*15:18	69TNT, 71TTS, 76ESN, 80N, 131S, 163LW	69TNT, 71TTS, 76ESN, 80N, 131S, 163LW
B*15:18	B*35:01, B*35:02, B*35:03, B*35:05, B*35:08, B*78:01	B*15:18 + 44RT + 193PV	69TNT, 71TTS, 76ESN, 80N, 131S, 163LW	44RT, 69TNT, 71TTS, 76ESN, 80N, 131S, 163LW, 193PV
B*15:18	B*50:01	B*15:18 + 41T + 45KE	69TNT, 71TTS, 76ESN, 80N, 131S, 163LW	41T, 45KE, 69TNT, 71TTS, 76ESN, 80N, 131S, 163LW
B*18:01	B*18:01, B*18:02	B*18:01	44RT, 69TNT, 71TTS, 76ESN, 80N, 131S	44RT, 69TNT, 71TTS, 76ESN, 80N, 131S
B*18:01	B*35:01, B*35:02, B*35:03, B*35:05, B*35:08, B*78:01	B*18:01 + 163LW + 193PV	44RT, 69TNT, 71TTS, 76ESN, 80N, 131S	44RT, 69TNT, 71TTS, 76ESN, 80N, 131S, 163LW, 193PV
B*27:03	B*27:03, B*27:05	B*27:03	65QIA, 69AA, 71ATD, 80TLR, 82LR, 131S, 163EW	65QIA, 69AA, 71ATD, 80TLR, 82LR, 131S, 163EW

B*27:05	B*27:03, B*27:05	B*27:05	65QIA, 69AA, 71ATD, 80TLR, 82LR, 131S, 163EW	65QIA, 69AA, 71ATD, 80TLR, 82LR, 131S, 163EW
B*27:08	B*27:08	B*27:08	65QIA, 69AA, 76ESN, 80N, 131S, 163EW	65QIA, 69AA, 76ESN, 80N, 131S, 163EW
B*35:01	B*35:01, B*35:02, B*35:03, B*35:05, B*35:08, B*78:01	B*35:01	44RT, 69TNT, 71TTS, 76ESN, 80N, 131S, 163LW, 193PV	44RT, 69TNT, 71TTS, 76ESN, 80N, 131S, 163LW, 193PV
B*35:08	B*35:01, B*35:02, B*35:03, B*35:05, B*35:08, B*78:01	B*35:08	44RT, 69TNT, 71TTS, 76ESN, 80N, 131S, 163LW, 193PV	44RT, 69TNT, 71TTS, 76ESN, 80N, 131S, 163LW, 193PV
B*37:01	B*37:01	B*37:01	44RT, 69TNT, 80TLR, 82LR, 131S, 156DA	44RT, 69TNT, 80TLR, 82LR, 131S, 156DA
B*38:01	B*38:01	B*38:01	69TNT, 80I, 82LR, 131S, 158T	69TNT, 80I, 82LR, 131S, 158T
B*39:01	B*39:01, B*39:05, B*39:09, B*39:15	B*39:01	69TNT, 71TTS, 76ESN, 80N, 131S, 158T	69TNT, 71TTS, 76ESN, 80N, 131S, 158T
B*40:01	B*40:01	B*40:01	41T, 45KE, 69TNT, 71TTS, 76ESN, 80N, 143S, 163EW, 180E	41T, 45KE, 69TNT, 71TTS, 76ESN, 80N, 143S, 163EW, 180E
B*40:02	B*40:01	B*40:02 + 143S + 180E	41T, 45KE, 69TNT, 71TTS, 76ESN, 80N, 163EW	41T, 45KE, 69TNT, 71TTS, 76ESN, 80N, 143S, 163EW, 180E
B*40:02	B*40:02, B*40:03, B*40:06	B*40:02	41T, 45KE, 69TNT, 71TTS, 76ESN, 80N, 163EW	41T, 45KE, 69TNT, 71TTS, 76ESN, 80N, 163EW
B*41:01	B*41:01	B*41:01	41T, 45KE, 69TNT, 71TTS, 76ESN, 80N, 156DA, 180E	41T, 45KE, 69TNT, 71TTS, 76ESN, 80N, 156DA, 180E
B*42:01	B*42:01	B*42:01	65QIA, 69AA, 70IAQ, 76ESN, 80N, 156DA, 180E	65QIA, 69AA, 70IAQ, 76ESN, 80N, 156DA, 180E
B*44:02	B*44:02	B*44:02	41T, 45KE, 69TNT, 80TLR, 82LR, 131S, 156DA, 163LS/G	41T, 45KE, 69TNT, 80TLR, 82LR, 131S, 156DA, 163LS/G
B*44:03	B*44:02	B*44:03 + 156DA	41T, 45KE, 69TNT, 80TLR, 82LR, 131S, 163LS/G	41T, 45KE, 69TNT, 80TLR, 82LR, 131S, 156DA, 163LS/G
B*44:03	B*44:03	B*44:03	41T, 45KE, 69TNT, 80TLR, 82LR, 131S, 163LS/G	41T, 45KE, 69TNT, 80TLR, 82LR, 131S, 163LS/G

B*45:01	B*45:01	B*45:01	41T, 45KE, 69TNT, 71TTS, 76ESN, 80N, 131S, 156DA, 163LS/G	41T, 45KE, 69TNT, 71TTS, 76ESN, 80N, 131S, 156DA, 163LS/G
B*46:01	B*46:01	B*46:01	44RMA, 65QKR, 73TVS, 76VRN, 80N, 131S, 163LW	44RMA, 65QKR, 73TVS, 76VRN, 80N, 131S, 163LW
B*47:01	B*47:01	B*47:01	41T, 45KE, 69TNT, 80TLR, 82LR, 131S, 163EW	41T, 45KE, 69TNT, 80TLR, 82LR, 131S, 163EW
B*48:01	B*40:01	B*48:01 + 41T + 45KE	69TNT, 71TTS, 76ESN, 80N, 143S, 163EW, 180E	41T, 45KE, 69TNT, 71TTS, 76ESN, 80N, 143S, 163EW, 180E
B*48:01	B*48:01, B*48:03	B*48:01	69TNT, 71TTS, 76ESN, 80N, 143S, 163EW, 180E	69TNT, 71TTS, 76ESN, 80N, 143S, 163EW, 180E
B*49:01	B*49:01	B*49:01	41T, 45KE, 69TNT, 80I, 82LR, 131S, 163LW	41T, 45KE, 69TNT, 80I, 82LR, 131S, 163LW
B*50:01	B*50:01	B*50:01	41T, 45KE, 69TNT, 71TTS, 76ESN, 80N, 131S, 163LW	41T, 45KE, 69TNT, 71TTS, 76ESN, 80N, 131S, 163LW
B*51:01	B*51:01, B*51:02, B*51:07, B*52:01, B*53:01	B*51:01	44RT, 69TNT, 80I, 82LR, 131S, 163LW, 193PV	44RT, 69TNT, 80I, 82LR, 131S, 163LW, 193PV
B*52:01	B*51:01, B*51:02, B*51:07, B*52:01, B*53:01	B*52:01	44RT, 69TNT, 80I, 82LR, 131S, 163LW, 193PV	44RT, 69TNT, 80I, 82LR, 131S, 163LW, 193PV
B*53:01	B*51:01, B*51:02, B*51:07, B*52:01, B*53:01	B*53:01	44RT, 69TNT, 80I, 82LR, 131S, 163LW, 193PV	44RT, 69TNT, 80I, 82LR, 131S, 163LW, 193PV
B*54:01	B*54:01, B*55:01, B*55:02	B*54:01	65QIA, 69AA, 70IAQ, 76ESN, 80N, 131S	65QIA, 69AA, 70IAQ, 76ESN, 80N, 131S
B*54:01	B*56:01, B*56:03, B*56:04	B*54:01 + 163LW	65QIA, 69AA, 70IAQ, 76ESN, 80N, 131S	65QIA, 69AA, 70IAQ, 76ESN, 80N, 131S, 163LW
B*54:01	B*67:01	B*54:01 + 158T	65QIA, 69AA, 70IAQ, 76ESN, 80N, 131S	65QIA, 69AA, 70IAQ, 76ESN, 80N, 131S, 158T
B*54:01	B*82:02	B*54:01 + 156DA + 163LS/G	65QIA, 69AA, 70IAQ, 76ESN, 80N, 131S	65QIA, 69AA, 70IAQ, 76ESN, 80N, 131S, 156DA, 163LS/G
B*55:01	B*54:01, B*55:01, B*55:02	B*55:01	65QIA, 69AA, 70IAQ, 76ESN, 80N, 131S	65QIA, 69AA, 70IAQ, 76ESN, 80N, 131S

B*55:01	B*56:01, B*56:03, B*56:04	B*55:01 + 163LW	65QIA, 69AA, 70IAQ, 76ESN, 80N, 131S	65QIA, 69AA, 70IAQ, 76ESN, 80N, 131S, 163LW
B*55:01	B*67:01	B*55:01 + 158T	65QIA, 69AA, 70IAQ, 76ESN, 80N, 131S	65QIA, 69AA, 70IAQ, 76ESN, 80N, 131S, 158T
B*55:01	B*82:02	B*55:01 + 156DA + 163LS/G	65QIA, 69AA, 70IAQ, 76ESN, 80N, 131S	65QIA, 69AA, 70IAQ, 76ESN, 80N, 131S, 156DA, 163LS/G
B*56:01	B*56:01, B*56:03, B*56:04	B*56:01	65QIA, 69AA, 70IAQ, 76ESN, 80N, 131S, 163LW	65QIA, 69AA, 70IAQ, 76ESN, 80N, 131S, 163LW
B*57:01	B*57:01	B*57:01	44RMA, 62GE, 62GRN, 65RNA, 69AA, 71SA, 80I, 82LR, 131S, 163LW	44RMA, 62GE, 62GRN, 65RNA, 69AA, 71SA, 80I, 82LR, 131S, 163LW
B*58:01	B*58:01	B*58:01	44RT, 62GE, 62GRN, 65RNA, 69AA, 71SA, 80I, 82LR, 131S, 163LW, 193PV	44RT, 62GE, 62GRN, 65RNA, 69AA, 71SA, 80I, 82LR, 131S, 163LW, 193PV
B*59:01	B*15:13	B*59:01 + 44RMA + 163LW	69TNT, 80I, 82LR, 131S	44RMA, 69TNT, 80I, 82LR, 131S, 163LW
B*59:01	B*38:01	B*59:01 + 158T	69TNT, 80I, 82LR, 131S	69TNT, 80I, 82LR, 131S, 158T
B*59:01	B*49:01	B*59:01 + 41T + 45KE + 163LW	69TNT, 80I, 82LR, 131S	41T, 45KE, 69TNT, 80I, 82LR, 131S, 163LW
B*59:01	B*51:01, B*51:02, B*51:07, B*52:01, B*53:01	B*59:01 + 44RT + 163LW + 193PV	69TNT, 80I, 82LR, 131S	44RT, 69TNT, 80I, 82LR, 131S, 163LW, 193PV
B*59:01	B*59:01	B*59:01	69TNT, 80I, 82LR, 131S	69TNT, 80I, 82LR, 131S
B*67:01	B*67:01	B*67:01	65QIA, 69AA, 70IAQ, 76ESN, 80N, 131S, 158T	65QIA, 69AA, 70IAQ, 76ESN, 80N, 131S, 158T
B*73:01	B*73:01	B*73:01	65QIA, 69AA, 76VRN, 80N, 90D, 163EW, 253Q, 267QE	65QIA, 69AA, 76VRN, 80N, 90D, 163EW, 253Q, 267QE
B*78:01	B*35:01, B*35:02, B*35:03, B*35:05, B*35:08, B*78:01	B*78:01	44RT, 69TNT, 71TTS, 76ESN, 80N, 131S, 163LW, 193PV	44RT, 69TNT, 71TTS, 76ESN, 80N, 131S, 163LW, 193PV
B*81:01	B*81:01	B*81:01	65QIA, 69AA, 70IAQ, 76ESN, 80N, 143S, 163EW, 180E	65QIA, 69AA, 70IAQ, 76ESN, 80N, 143S, 163EW, 180E
B*82:02	B*82:02	B*82:02	65QIA, 69AA, 70IAQ, 76ESN, 80N, 131S, 156DA, 163LS/G	65QIA, 69AA, 70IAQ, 76ESN, 80N, 131S, 156DA, 163LS/G

C*01:02	C*01:02, C*01:03	C*01:02	65QKR, 73TVS, 76VRN, 80N, 193PV, 219W, 248M	65QKR, 73TVS, 76VRN, 80N, 193PV, 219W, 248M
C*02:02	C*02:02	C*02:02	21H, 65QKR, 80K, 163EW, 193PV	21H, 65QKR, 80K, 163EW, 193PV
C*03:03	C*03:02, C*03:03, C*03:04, C*03:17	C*03:03	21H, 65QKR, 73TVS, 76VRN, 80N, 163LW, 173K, 193PV, 219W	21H, 65QKR, 73TVS, 76VRN, 80N, 163LW, 173K, 193PV, 219W
C*03:04	C*03:02, C*03:03, C*03:04, C*03:17	C*03:04	21H, 65QKR, 73TVS, 76VRN, 80N, 163LW, 173K, 193PV, 219W	21H, 65QKR, 73TVS, 76VRN, 80N, 163LW, 173K, 193PV, 219W
C*04:01	C*04:01, C*18:01	C*04:01	65QKR, 73AN, 80K, 90D, 193PV, 219W	65QKR, 73AN, 80K, 90D, 193PV, 219W
C*04:01	C*04:03, C*04:06	C*04:01 + 21H	65QKR, 73AN, 80K, 90D, 193PV, 219W	21H, 65QKR, 73AN, 80K, 90D, 193PV, 219W
C*04:03	C*04:03, C*04:06	C*04:03	21H, 65QKR, 73AN, 80K, 90D, 193PV, 219W	21H, 65QKR, 73AN, 80K, 90D, 193PV, 219W
C*05:01	C*05:01	C*05:01	65QKR, 80K, 138K, 177KT, 193PV	65QKR, 80K, 138K, 177KT, 193PV
C*06:02	C*04:01, C*18:01	C*06:02 + 219W	65QKR, 73AN, 80K, 90D, 193PV	65QKR, 73AN, 80K, 90D, 193PV, 219W
C*06:02	C*04:03, C*04:06	C*06:02 + 21H + 219W	65QKR, 73AN, 80K, 90D, 193PV	21H, 65QKR, 73AN, 80K, 90D, 193PV, 219W
C*06:02	C*06:02	C*06:02	65QKR, 73AN, 80K, 90D, 193PV	65QKR, 73AN, 80K, 90D, 193PV
C*07:01	C*07:01, C*07:06	C*07:01	76VRN, 80N, 90D, 193PL, 253Q, 267QE	76VRN, 80N, 90D, 193PL, 253Q, 267QE
C*07:01	C*07:02, C*07:29	C*07:01 + 65QKR	76VRN, 80N, 90D, 193PL, 253Q, 267QE	65QKR, 76VRN, 80N, 90D, 193PL, 253Q, 267QE
C*07:01	C*07:04	C*07:01 + 65QKR + 156DA + 177KT	76VRN, 80N, 90D, 193PL, 253Q, 267QE	65QKR, 76VRN, 80N, 90D, 156DA, 177KT, 193PL, 253Q, 267QE
C*07:02	C*07:02, C*07:29	C*07:02	65QKR, 76VRN, 80N, 90D, 193PL, 253Q, 267QE	65QKR, 76VRN, 80N, 90D, 193PL, 253Q, 267QE
C*07:02	C*07:04	C*07:02 + 156DA + 177KT	65QKR, 76VRN, 80N, 90D, 193PL, 253Q, 267QE	65QKR, 76VRN, 80N, 90D, 156DA, 177KT, 193PL, 253Q, 267QE
C*08:01	C*08:01, C*08:03	C*08:01	65QKR, 73TVS, 76VRN, 80N, 177KT, 193PV	65QKR, 73TVS, 76VRN, 80N, 177KT, 193PV
C*08:01	C*08:02, C*08:04	C*08:01 + 138K	65QKR, 73TVS, 76VRN, 80N, 177KT, 193PV	65QKR, 73TVS, 76VRN, 80N, 138K, 177KT, 193PV

C*08:02	C*08:02, C*08:04	C*08:02	65QKR, 73TVS, 76VRN, 80N, 138K, 177KT, 193PV	65QKR, 73TVS, 76VRN, 80N, 138K, 177KT, 193PV
C*12:02	C*01:02, C*01:03	C*12:02 + 73TVS + 219W + 248M	65QKR, 76VRN, 80N, 193PV	65QKR, 73TVS, 76VRN, 80N, 193PV, 219W, 248M
C*12:02	C*03:02, C*03:03, C*03:04, C*03:17	C*12:02 + 21H + 73TVS + 163LW + 173K + 219W	65QKR, 76VRN, 80N, 193PV	21H, 65QKR, 73TVS, 76VRN, 80N, 163LW, 173K, 193PV, 219W
C*12:02	C*08:01, C*08:03	C*12:02 + 73TVS + 177KT	65QKR, 76VRN, 80N, 193PV	65QKR, 73TVS, 76VRN, 80N, 177KT, 193PV
C*12:02	C*08:02, C*08:04	C*12:02 + 73TVS + 138K + 177KT	65QKR, 76VRN, 80N, 193PV	65QKR, 73TVS, 76VRN, 80N, 138K, 177KT, 193PV
C*12:02	C*12:02, C*12:03	C*12:02	65QKR, 76VRN, 80N, 193PV	65QKR, 76VRN, 80N, 193PV
C*12:02	C*14:02	C*12:02 + 73TVS + 219W	65QKR, 76VRN, 80N, 193PV	65QKR, 73TVS, 76VRN, 80N, 193PV, 219W
C*12:02	C*14:03	C*12:02 + 21H + 73TVS + 219W	65QKR, 76VRN, 80N, 193PV	21H, 65QKR, 73TVS, 76VRN, 80N, 193PV, 219W
C*14:02	C*01:02, C*01:03	C*14:02 + 248M	65QKR, 73TVS, 76VRN, 80N, 193PV, 219W	65QKR, 73TVS, 76VRN, 80N, 193PV, 219W, 248M
C*14:02	C*03:02, C*03:03, C*03:04, C*03:17	C*14:02 + 21H + 163LW + 173K	65QKR, 73TVS, 76VRN, 80N, 193PV, 219W	21H, 65QKR, 73TVS, 76VRN, 80N, 163LW, 173K, 193PV, 219W
C*14:02	C*14:02	C*14:02	65QKR, 73TVS, 76VRN, 80N, 193PV, 219W	65QKR, 73TVS, 76VRN, 80N, 193PV, 219W
C*14:02	C*14:03	C*14:02 + 21H	65QKR, 73TVS, 76VRN, 80N, 193PV, 219W	21H, 65QKR, 73TVS, 76VRN, 80N, 193PV, 219W
C*15:02	C*02:02	C*15:02 + 65QKR + 163EW	21H, 80K, 193PV	21H, 65QKR, 80K, 163EW, 193PV
C*15:02	C*04:03, C*04:06	C*15:02 + 65QKR + 73AN + 90D + 219W	21H, 80K, 193PV	21H, 65QKR, 73AN, 80K, 90D, 193PV, 219W
C*15:02	C*15:02, C*15:05	C*15:02	21H, 80K, 193PV	21H, 80K, 193PV
C*16:01	B*46:01	C*16:01 + 44RMA + 131S + 163LW	65QKR, 73TVS, 76VRN, 80N	44RMA, 65QKR, 73TVS, 76VRN, 80N, 131S, 163LW

C*16:01	C*01:02, C*01:03	C*16:01 + 193PV + 219W + 248M	65QKR, 73TVS, 76VRN, 80N	65QKR, 73TVS, 76VRN, 80N, 193PV, 219W, 248M
C*16:01	C*03:02, C*03:03, C*03:04, C*03:17	C*16:01 + 21H + 163LW + 173K + 193PV + 219W	65QKR, 73TVS, 76VRN, 80N	21H, 65QKR, 73TVS, 76VRN, 80N, 163LW, 173K, 193PV, 219W
C*16:01	C*08:01, C*08:03	C*16:01 + 177KT + 193PV	65QKR, 73TVS, 76VRN, 80N	65QKR, 73TVS, 76VRN, 80N, 177KT, 193PV
C*16:01	C*08:02, C*08:04	C*16:01 + 138K + 177KT + 193PV	65QKR, 73TVS, 76VRN, 80N	65QKR, 73TVS, 76VRN, 80N, 138K, 177KT, 193PV
C*16:01	C*14:02	C*16:01 + 193PV + 219W	65QKR, 73TVS, 76VRN, 80N	65QKR, 73TVS, 76VRN, 80N, 193PV, 219W
C*16:01	C*14:03	C*16:01 + 21H + 193PV + 219W	65QKR, 73TVS, 76VRN, 80N	21H, 65QKR, 73TVS, 76VRN, 80N, 193PV, 219W
C*16:01	C*16:01, C*16:04	C*16:01	65QKR, 73TVS, 76VRN, 80N	65QKR, 73TVS, 76VRN, 80N
C*17:01	C*17:01	C*17:01	65QKR, 73AN, 80K, 143S, 163EW, 193PV, 253Q, 267QE	65QKR, 73AN, 80K, 143S, 163EW, 193PV, 253Q, 267QE
C*18:01	C*04:01, C*18:01	C*18:01	65QKR, 73AN, 80K, 90D, 193PV, 219W	65QKR, 73AN, 80K, 90D, 193PV, 219W
C*18:01	C*04:03, C*04:06	C*18:01 + 21H	65QKR, 73AN, 80K, 90D, 193PV, 219W	21H, 65QKR, 73AN, 80K, 90D, 193PV, 219W
DQB1*02:01	DQB1*02:01, DQB1*02:02	DQB1*02:01	52LL, 77R, 84QL, 182S, rq70RK/R	52LL, 77R, 84QL, 182S, rq70RK/R
DQB1*02:02	DQB1*02:01, DQB1*02:02	DQB1*02:02	52LL, 77R, 84QL, 182S, rq70RK/R	52LL, 77R, 84QL, 182S, rq70RK/R
DQB1*03:01	DQB1*03:01	DQB1*03:01	45EV, 46VY, 55PP, 77T, 84QL, 182N	45EV, 46VY, 55PP, 77T, 84QL, 182N
DQB1*03:02	DQB1*03:02, DQB1*03:03, DQB1*03:05	DQB1*03:02	45GV, 46VY, 55PP, 77T, 84QL, 182N	45GV, 46VY, 55PP, 77T, 84QL, 182N
DQB1*03:03	DQB1*03:02, DQB1*03:03, DQB1*03:05	DQB1*03:03	45GV, 46VY, 55PP, 77T, 84QL, 182N	45GV, 46VY, 55PP, 77T, 84QL, 182N
DQB1*04:01	DQB1*04:01, DQB1*04:02	DQB1*04:01	45GV, 46VY, 55R, 56L, 74S, 77T, 84QL, 182N, rq75VT	45GV, 46VY, 55R, 56L, 74S, 77T, 84QL, 182N, rq75VT
DQB1*04:02	DQB1*04:01, DQB1*04:02	DQB1*04:02	45GV, 46VY, 55R, 56L, 74S, 77T, 84QL, 182N, rq75VT	45GV, 46VY, 55R, 56L, 74S, 77T, 84QL, 182N, rq75VT

DQB1*05:01	DQB1*05:01	DQB1*05:01	45GV, 46VY, 52PQ, 55R, 57V, 74S, 77R, 87Y, 116I, 125SQ, 182S	45GV, 46VY, 52PQ, 55R, 57V, 74S, 77R, 87Y, 116I, 125SQ, 182S
DQB1*05:02	DQB1*05:01	DQB1*05:02 + 57V + 125SQ	45GV, 46VY, 52PQ, 55R, 74S, 77R, 87Y, 116I, 182S	45GV, 46VY, 52PQ, 55R, 57V, 74S, 77R, 87Y, 116I, 125SQ, 182S
DQB1*05:02	DQB1*05:02	DQB1*05:02	45GV, 46VY, 52PQ, 55R, 74S, 77R, 87Y, 116I, 182S	45GV, 46VY, 52PQ, 55R, 74S, 77R, 87Y, 116I, 182S
DQB1*05:02	DQB1*05:03	DQB1*05:02 + 125SQ	45GV, 46VY, 52PQ, 55R, 74S, 77R, 87Y, 116I, 182S	45GV, 46VY, 52PQ, 55R, 74S, 77R, 87Y, 116I, 125SQ, 182S
DQB1*05:03	DQB1*05:01	DQB1*05:03 + 57V	45GV, 46VY, 52PQ, 55R, 74S, 77R, 87Y, 116I, 125SQ, 182S	45GV, 46VY, 52PQ, 55R, 57V, 74S, 77R, 87Y, 116I, 125SQ, 182S
DQB1*05:03	DQB1*05:03	DQB1*05:03	45GV, 46VY, 52PQ, 55R, 74S, 77R, 87Y, 116I, 125SQ, 182S	45GV, 46VY, 52PQ, 55R, 74S, 77R, 87Y, 116I, 125SQ, 182S
DQB1*06:01	DQB1*06:01, DQB1*06:02, DQB1*06:03, DQB1*06:10, DQB1*06:11	DQB1*06:01	45GV, 46VY, 52PQ, 55R, 77T, 87F, 182S	45GV, 46VY, 52PQ, 55R, 77T, 87F, 182S
DQB1*06:02	DQB1*06:01, DQB1*06:02, DQB1*06:03, DQB1*06:10, DQB1*06:11	DQB1*06:02	45GV, 46VY, 52PQ, 55R, 77T, 87F, 182S	45GV, 46VY, 52PQ, 55R, 77T, 87F, 182S
DQB1*06:03	DQB1*06:01, DQB1*06:02, DQB1*06:03, DQB1*06:10, DQB1*06:11	DQB1*06:03	45GV, 46VY, 52PQ, 55R, 77T, 87F, 182S	45GV, 46VY, 52PQ, 55R, 77T, 87F, 182S
DQB1*06:04	DQB1*06:04, DQB1*06:05, DQB1*06:09	DQB1*06:04	45GV, 46VY, 52PQ, 55R, 57V, 77T, 87Y, 182S	45GV, 46VY, 52PQ, 55R, 57V, 77T, 87Y, 182S
DRB1*01:01	DRB1*01:01, DRB1*01:02	DRB1*01:01	4R, 13FE, 25R, 30C, 67LQ, 70QT, 73A, 77T, 96EV, rq75VT	4R, 13FE, 25R, 30C, 67LQ, 70QT, 73A, 77T, 96EV, rq75VT
DRB1*01:02	DRB1*01:01, DRB1*01:02	DRB1*01:02	4R, 13FE, 25R, 30C, 67LQ, 70QT, 73A, 77T, 96EV, rq75VT	4R, 13FE, 25R, 30C, 67LQ, 70QT, 73A, 77T, 96EV, rq75VT
DRB1*01:03	DRB1*01:03	DRB1*01:03	4R, 13FE, 25R, 30C, 70D, 70DA, 73A, 77T, 96EV, rp67IE, rq75VT	4R, 13FE, 25R, 30C, 70D, 70DA, 73A, 77T, 96EV, rp67IE, rq75VT

DRB1*03:01	DRB1*03:01	DRB1*03:01	4R, 11STS, 25R, 47F, 67LQ, 74R, 77N, 96HK	4R, 11STS, 25R, 47F, 67LQ, 74R, 77N, 96HK
DRB1*03:02	DRB1*03:01	DRB1*03:02 + 47F	4R, 11STS, 25R, 67LQ, 74R, 77N, 96HK	4R, 11STS, 25R, 47F, 67LQ, 74R, 77N, 96HK
DRB1*03:02	DRB1*03:02	DRB1*03:02	4R, 11STS, 25R, 67LQ, 74R, 77N, 96HK	4R, 11STS, 25R, 67LQ, 74R, 77N, 96HK
DRB1*03:03	DRB1*03:01	DRB1*03:03 + 4R + 47F	11STS, 25R, 67LQ, 74R, 77N, 96HK	4R, 11STS, 25R, 47F, 67LQ, 74R, 77N, 96HK
DRB1*03:03	DRB1*03:02	DRB1*03:03 + 4R	11STS, 25R, 67LQ, 74R, 77N, 96HK	4R, 11STS, 25R, 67LQ, 74R, 77N, 96HK
DRB1*03:03	DRB1*03:03	DRB1*03:03	11STS, 25R, 67LQ, 74R, 77N, 96HK	11STS, 25R, 67LQ, 74R, 77N, 96HK
DRB1*04:01	DRB1*04:01, DRB1*04:03, DRB1*04:04, DRB1*04:07	DRB1*04:01	4R, 25R, 37YV, 67LQ, 70QT, 73A, 77T, 96Y, 98E, 104A, rq75VT	4R, 25R, 37YV, 67LQ, 70QT, 73A, 77T, 96Y, 98E, 104A, rq75VT
DRB1*04:01	DRB1*04:05, DRB1*04:10	DRB1*04:01 + 57S	4R, 25R, 37YV, 67LQ, 70QT, 73A, 77T, 96Y, 98E, 104A, rq75VT	4R, 25R, 37YV, 57S, 67LQ, 70QT, 73A, 77T, 96Y, 98E, 104A, rq75VT
DRB1*04:02	DRB1*04:02	DRB1*04:02	4R, 25R, 37YV, 70D, 70DA, 73A, 77T, 96Y, 98E, 104A, rp67IE, rq75VT	4R, 25R, 37YV, 70D, 70DA, 73A, 77T, 96Y, 98E, 104A, rp67IE, rq75VT
DRB1*04:03	DRB1*04:01, DRB1*04:03, DRB1*04:04, DRB1*04:07	DRB1*04:03	4R, 25R, 37YV, 67LQ, 70QT, 73A, 77T, 96Y, 98E, 104A, rq75VT	4R, 25R, 37YV, 67LQ, 70QT, 73A, 77T, 96Y, 98E, 104A, rq75VT
DRB1*04:03	DRB1*04:05, DRB1*04:10	DRB1*04:03 + 57S	4R, 25R, 37YV, 67LQ, 70QT, 73A, 77T, 96Y, 98E, 104A, rq75VT	4R, 25R, 37YV, 57S, 67LQ, 70QT, 73A, 77T, 96Y, 98E, 104A, rq75VT
DRB1*04:04	DRB1*04:01, DRB1*04:03, DRB1*04:04, DRB1*04:07	DRB1*04:04	4R, 25R, 37YV, 67LQ, 70QT, 73A, 77T, 96Y, 98E, 104A, rq75VT	4R, 25R, 37YV, 67LQ, 70QT, 73A, 77T, 96Y, 98E, 104A, rq75VT
DRB1*04:04	DRB1*04:05, DRB1*04:10	DRB1*04:04 + 57S	4R, 25R, 37YV, 67LQ, 70QT, 73A, 77T, 96Y, 98E, 104A, rq75VT	4R, 25R, 37YV, 57S, 67LQ, 70QT, 73A, 77T, 96Y, 98E, 104A, rq75VT
DRB1*04:05	DRB1*04:05, DRB1*04:10	DRB1*04:05	4R, 25R, 37YV, 57S, 67LQ, 70QT, 73A, 77T, 96Y, 98E, 104A, rq75VT	4R, 25R, 37YV, 57S, 67LQ, 70QT, 73A, 77T, 96Y, 98E, 104A, rq75VT
DRB1*07:01	DRB1*07:01	DRB1*07:01	4Q, 25Q, 57V, 70D, 77T, 98E, 98ES, 104A, 181M, rq75VT	4Q, 25Q, 57V, 70D, 77T, 98E, 98ES, 104A, 181M, rq75VT
DRB1*08:01	DRB1*08:01, DRB1*08:03	DRB1*08:01	4R, 16Y, 25R, 37YV, 57S, 70D, 70DA, 73A, 77T, 96HK, rq75VT	4R, 16Y, 25R, 37YV, 57S, 70D, 70DA, 73A, 77T, 96HK, rq75VT

DRB1*08:02	DRB1*08:01, DRB1*08:03	DRB1*08:02 + 57S	4R, 16Y, 25R, 37YV, 70D, 70DA, 73A, 77T, 96HK, rq75VT	4R, 16Y, 25R, 37YV, 57S, 70D, 70DA, 73A, 77T, 96HK, rq75VT
DRB1*08:02	DRB1*08:02	DRB1*08:02	4R, 16Y, 25R, 37YV, 70D, 70DA, 73A, 77T, 96HK, rq75VT	4R, 16Y, 25R, 37YV, 70D, 70DA, 73A, 77T, 96HK, rq75VT
DRB1*09:01	DRB1*09:01	DRB1*09:01	4Q, 13FE, 25R, 57V, 70R, 73A, 77T, 98E, 98ES, 104A, 181M, rq70RK/R, rq75VT	4Q, 13FE, 25R, 57V, 70R, 73A, 77T, 98E, 98ES, 104A, 181M, rq70RK/R, rq75VT
DRB1*10:01	DRB1*10:01	DRB1*10:01	4R, 13FE, 25R, 30RV, 70R, 73A, 77T, 181M, rq70RK/R, rq75VT	4R, 13FE, 25R, 30RV, 70R, 73A, 77T, 181M, rq70RK/R, rq75VT
DRB1*11:01	DRB1*11:01, DRB1*11:03, DRB1*11:04, DRB1*11:06	DRB1*11:01	4R, 11STS, 25R, 37YV, 47F, 57DE, 70D, 70DA, 73A, 77T, 96HK, rp58E, rp58EE, rq75VT	4R, 11STS, 25R, 37YV, 47F, 57DE, 70D, 70DA, 73A, 77T, 96HK, rp58E, rp58EE, rq75VT
DRB1*11:03	DRB1*11:01, DRB1*11:03, DRB1*11:04, DRB1*11:06	DRB1*11:03	4R, 11STS, 25R, 37YV, 47F, 57DE, 70D, 70DA, 73A, 77T, 96HK, rp58E, rp58EE, rq75VT	4R, 11STS, 25R, 37YV, 47F, 57DE, 70D, 70DA, 73A, 77T, 96HK, rp58E, rp58EE, rq75VT
DRB1*11:04	DRB1*11:01, DRB1*11:03, DRB1*11:04, DRB1*11:06	DRB1*11:04	4R, 11STS, 25R, 37YV, 47F, 57DE, 70D, 70DA, 73A, 77T, 96HK, rp58E, rp58EE, rq75VT	4R, 11STS, 25R, 37YV, 47F, 57DE, 70D, 70DA, 73A, 77T, 96HK, rp58E, rp58EE, rq75VT
DRB1*12:01	DRB1*12:01, DRB1*12:02	DRB1*12:01	4R, 16Y, 25R, 37L, 47F, 57V, 70D, 70DA, 73A, 77T, 96HK, rq75VT	4R, 16Y, 25R, 37L, 47F, 57V, 70D, 70DA, 73A, 77T, 96HK, rq75VT
DRB1*12:02	DRB1*12:01, DRB1*12:02	DRB1*12:02	4R, 16Y, 25R, 37L, 47F, 57V, 70D, 70DA, 73A, 77T, 96HK, rq75VT	4R, 16Y, 25R, 37L, 47F, 57V, 70D, 70DA, 73A, 77T, 96HK, rq75VT
DRB1*13:01	DRB1*13:01, DRB1*13:02	DRB1*13:01	4R, 11STS, 25R, 47F, 70D, 70DA, 73A, 77T, 96HK, rp67IE, rq75VT	4R, 11STS, 25R, 47F, 70D, 70DA, 73A, 77T, 96HK, rp67IE, rq75VT
DRB1*13:03	DRB1*13:03, DRB1*13:12	DRB1*13:03	4R, 11STS, 25R, 37YV, 57S, 70D, 70DA, 73A, 77T, 96HK, rq75VT	4R, 11STS, 25R, 37YV, 57S, 70D, 70DA, 73A, 77T, 96HK, rq75VT
DRB1*13:05	DRB1*11:01, DRB1*11:03, DRB1*11:04, DRB1*11:06	DRB1*13:05 + 37YV + 57DE + rp58E + rp58EE	4R, 11STS, 25R, 47F, 70D, 70DA, 73A, 77T, 96HK, rq75VT	4R, 11STS, 25R, 37YV, 47F, 57DE, 70D, 70DA, 73A, 77T, 96HK, rp58E, rp58EE, rq75VT
DRB1*13:05	DRB1*13:01, DRB1*13:02	DRB1*13:05 + rp67IE	4R, 11STS, 25R, 47F, 70D, 70DA, 73A, 77T, 96HK, rq75VT	4R, 11STS, 25R, 47F, 70D, 70DA, 73A, 77T, 96HK, rp67IE, rq75VT

DRB1*13:05	DRB1*13:05	DRB1*13:05	4R, 11STS, 25R, 47F, 70D, 70DA, 73A, 77T, 96HK, rq75VT	4R, 11STS, 25R, 47F, 70D, 70DA, 73A, 77T, 96HK, rq75VT
DRB1*14:01	DRB1*14:01, DRB1*14:05, DRB1*14:07, DRB1*14:54	DRB1*14:01	4R, 11STS, 25R, 70R, 73A, 77T, 96HK, rq70RK/R, rq75VT	4R, 11STS, 25R, 70R, 73A, 77T, 96HK, rq70RK/R, rq75VT
DRB1*14:03	DRB1*11:01, DRB1*11:03, DRB1*11:04, DRB1*11:06	DRB1*14:03 + 37YV + 47F + 57DE + rp58E + rp58EE	4R, 11STS, 25R, 70D, 70DA, 73A, 77T, 96HK, rq75VT	4R, 11STS, 25R, 37YV, 47F, 57DE, 70D, 70DA, 73A, 77T, 96HK, rp58E, rp58EE, rq75VT
DRB1*14:03	DRB1*13:01, DRB1*13:02	DRB1*14:03 + 47F + rp67IE	4R, 11STS, 25R, 70D, 70DA, 73A, 77T, 96HK, rq75VT	4R, 11STS, 25R, 47F, 70D, 70DA, 73A, 77T, 96HK, rp67IE, rq75VT
DRB1*14:03	DRB1*13:03, DRB1*13:12	DRB1*14:03 + 37YV + 57S	4R, 11STS, 25R, 70D, 70DA, 73A, 77T, 96HK, rq75VT	4R, 11STS, 25R, 37YV, 57S, 70D, 70DA, 73A, 77T, 96HK, rq75VT
DRB1*14:03	DRB1*13:05	DRB1*14:03 + 47F	4R, 11STS, 25R, 70D, 70DA, 73A, 77T, 96HK, rq75VT	4R, 11STS, 25R, 47F, 70D, 70DA, 73A, 77T, 96HK, rq75VT
DRB1*14:03	DRB1*14:03	DRB1*14:03	4R, 11STS, 25R, 70D, 70DA, 73A, 77T, 96HK, rq75VT	4R, 11STS, 25R, 70D, 70DA, 73A, 77T, 96HK, rq75VT
DRB1*14:04	DRB1*14:04	DRB1*14:04	4R, 16Y, 25R, 70R, 73A, 77T, 96HK, rq70RK/R, rq75VT	4R, 16Y, 25R, 70R, 73A, 77T, 96HK, rq70RK/R, rq75VT
DRB1*15:01	DRB1*15:01, DRB1*15:02, DRB1*15:03	DRB1*15:01	4R, 25R, 47F, 70QT, 73A, 77T, 142M, rq75VT	4R, 25R, 47F, 70QT, 73A, 77T, 142M, rq75VT
DRB1*15:02	DRB1*15:01, DRB1*15:02, DRB1*15:03	DRB1*15:02	4R, 25R, 47F, 70QT, 73A, 77T, 142M, rq75VT	4R, 25R, 47F, 70QT, 73A, 77T, 142M, rq75VT
DRB1*15:03	DRB1*15:01, DRB1*15:02, DRB1*15:03	DRB1*15:03	4R, 25R, 47F, 70QT, 73A, 77T, 142M, rq75VT	4R, 25R, 47F, 70QT, 73A, 77T, 142M, rq75VT
DRB1*16:01	DRB1*16:01, DRB1*16:02	DRB1*16:01	4R, 25R, 70D, 70DA, 73A, 77T, 142M, rq75VT	4R, 25R, 70D, 70DA, 73A, 77T, 142M, rq75VT
DRB1*16:02	DRB1*16:01, DRB1*16:02	DRB1*16:02	4R, 25R, 70D, 70DA, 73A, 77T, 142M, rq75VT	4R, 25R, 70D, 70DA, 73A, 77T, 142M, rq75VT

Table S2. Numbers of eplets that were shared by LSA antigens and their eplet-predicted antigens.

HLA locus	Min	Max	Mean	Mean Normalization*
A	5	11	8.31	0.551
C	3	9	5.16	0.360
B	4	8	6.02	0.504
DRB1	6	14	10.6	0.570
DQB1	5	10	6.93	0.387

* Normalization of mean = (Mean - Min) / (Max - Min), which were comparable between HLA loci.
